# Characteristics of Hydrogels as a Coating for Microneedle Transdermal Delivery Systems with Agomelatine

**DOI:** 10.3390/molecules30020322

**Published:** 2025-01-15

**Authors:** Monika Wojtyłko, Ariadna B. Nowicka, Anna Froelich, Mirosław Szybowicz, Tobiasz Banaszek, Dorota Tomczak, Wiesław Kuczko, Radosław Wichniarek, Irena Budnik, Barbara Jadach, Oliwia Kordyl, Antoni Białek, Julia Krysztofiak, Tomasz Osmałek, Dimitrios A. Lamprou

**Affiliations:** 1 3D Printing Division, Chair and Department of Pharmaceutical Technology, Poznan University of Medical Sciences, 3 Rokietnicka Street, 60-806 Poznań, Poland; mwojtylko@ump.edu.pl (M.W.); froelich@ump.edu.pl (A.F.); okordyl@ump.edu.pl (O.K.); 2 Doctoral School, Poznan University of Medical Sciences, 70 Bukowska Street, 60-812 Poznań, Poland; 3 Institute of Materials Research and Quantum Engineering, The Faculty of Materials Engineering and Technical Physics, Poznan University of Technology, 3 Piotrowo Street, 60-965 Poznań, Poland; ariadna.nowicka@put.poznan.pl (A.B.N.); miroslaw.szybowicz@put.poznan.pl (M.S.);; 4 Institute of Chemical Technology and Engineering, Poznan University of Technology, 4 Berdychowo Street, 60-965 Poznań, Poland; dorota.tomczak@doctorate.put.poznan.pl; 5 Institute of Materials Technology, Faculty of Mechanical Engineering, Poznan University of Technology, 3 Piotrowo Street, 60-965 Poznań, Poland; wieslaw.kuczko@put.poznan.pl (W.K.);; 6 Chair and Department of Pharmaceutical Technology, Poznan University of Medical Sciences, 3 Rokietnicka Street, 60-806 Poznań, Polandantoni.s.bialek@gmail.com (A.B.); julia.krysztofiak@icloud.com (J.K.); 7 Division of Industrial Pharmacy, Chair and Department of Pharmaceutical Technology, Poznan University of Medical Sciences, 3 Rokietnicka Street, 60-806 Poznań, Poland; bjadach@ump.edu.pl; 8 Student’s Research Group of Pharmaceutical Technology, The Student Scientific Society of Poznan University of Medical Sciences, 5 Rokietnicka Street, 60-806 Poznań, Poland; 9 School of Pharmacy, Queen’s University Belfast, Belfast BT9 7BL, UK

**Keywords:** agomelatine, transdermal delivery, hydrogel, coated microneedles

## Abstract

Agomelatine (AGM) is an effective antidepressant with low oral bioavailability due to intensive hepatic metabolism. Transdermal administration of agomelatine may increase its bioavailability and reduce the doses necessary for therapeutic effects. However, transdermal delivery requires crossing the *stratum corneum* barrier. For this purpose, the use of microneedles may increase the efficiency of administration. The aim of this study was to prepare an agomelatine-loaded hydrogel suitable for coating microneedles for the transdermal drug delivery of AGM. The optimized formulations were subjected to spectroscopic and rheological characterization and mechanical tests, as well as tested for release through an artificial membrane and permeation through human skin ex vivo. Both hydrogels were found to have suitable parameters for coating microneedles using the dip-coating method, including the stability of the substance at the process temperature, shear-thinning behavior, and appropriate textural parameters such as adhesion or hardness. Additionally, two formulations were tested for potential application to the skin alone because the gels showed suitable mechanical properties for the skin application. In this case, the ethanol gel was characterized by higher skin permeability and better spreadability. The information obtained in this study will allow the preparation of coated microneedles for the transdermal administration of agomelatine.

## 1. Introduction

Agomelatine (AGM, N-[2-(7-methoxy-1-naphthyl)ethyl]acetamide) is an atypical antidepressant drug that entered the European Union market in 2009. Its mechanism of action includes an agonist effect on melatonin receptors MT1 and MT2 and antagonism for serotonin receptors 5-HT_2C_ [1]. AGM is generally used in the treatment of major depressive disorders (MDDs) in adults. Although, through its activity on serotonin receptors, it also increases the level of norepinephrine and dopamine in the prefrontal cortex and influences the GABAergic pathway, which leads to antidepressant, nootropic, and anxiolytic effects [2,3]. Additionally, the agonism to melatonin receptors helps to resynchronize the circadian rhythm, which, in turn, benefits the treatment of MDDs [4]. Also, it was proven that agomelatine administered orally exerts a hypotonic effect on an intraocular pressure in the treatment of glaucoma [5].

Currently, the only dosage form of AGM available on the market is a conventional tablet. A daily dose of AGM is 25–50 mg for an adult, taken at bedtime. The substance is rapidly absorbed, reaching the maximum concentration in plasma within 1–2 h, and metabolized to non-active compounds by isoenzymes: CYP1A2 to the greatest extent and CYP2C9 and CYP2C19 to a lesser extent. However, the absolute bioavailability after oral administration is lower than 5% [6]. Moreover, there is significant variability in bioavailability among individuals, which is the result of the first-pass effect, liver condition, and differences in CYP1A2 isoenzyme activity [7].

An enhancement in AGM oral bioavailability became a subject of research. Administering the lower dose of the drug could potentially benefit in decreasing the risk of side effects. Also, omitting the first-pass effect could help reduce the interindividual differences in the bioavailability of AGM. Prajapati et al. [8] tried to avoid the first-pass effect by targeting lymphatic uptake. The authors presented AGM-loaded nanostructured lipid carriers (NLCs) that can be absorbed by the M cells of Peyer’s patches. However, taking into consideration the low bioavailability of AGM after oral administration, it also seems reasonable to look for an alternative route of administration. There have already been some attempts to deliver the drug intranasally using polymeric nanoparticles as a carrier [9] or mucoadhesive nanoemulsions obtained with egg lecithin [10]. Also, AGM-loaded poly (D, L-lactide-co-glycolide) (PLGA) microspheres with prolonged action were investigated for intramuscular injection [11]. Another option that could be beneficial in terms of higher bioavailability is the transdermal route of administration.

The concept of AGM transdermal delivery is quite new and, within the last few years, has been addressed only in a few studies. A common aim of the research is to deliver agomelatine by the transdermal route of administration and enhance the bioavailability of the drug using various approaches, such as the form of a microemulsion gel [12], application of invasomes enhanced by ultrasounds [13], or preparation of nanocarriers: polymeric nanoparticles [14], nanostructured lipid carriers [15], and liquid nanocrystals [16]. Also, Nemr et al. [17] prepared agomelatine-loaded bilosomes enriched with hyaluronic acid for topical application to the eye to lower the intraocular pressure in glaucoma. However, there is still a large field to explore in the matter of more efficient AGM delivery.

Besides nanocarriers and energy-driven methods such as ionophoresis and sonophoresis, permeation enhancers (e.g., alcohols, azone, surfactants) or methods bypassing the *stratum corneum* (SC) barrier are also being used to facilitate the transdermal delivery of drugs. Microneedles (MNs) belong to the latter category [18].

Microneedles are small (up to 2000 µm) needles with sharp tips that are able to create pores on the skin without causing bleeding or pain, which makes them superior to hypodermic injection in terms of patient compliance and comfort [19]. MNs can physically pierce the skin, overcoming the SC, which is the outermost layer of the epidermis, delivering the drug to the viable epidermis or dermis, where it could enter the systemic circulation through small vessels. Various types of MNs have been developed so far, with solid, hollow, dissolving, and coated MNs being the most popular types [20]. Coated MNs carry a cargo of the therapeutic agent that can be dissolved in the skin almost immediately or persist, providing a sustained release. This type of MN is characterized by good stability in a solid state and ease of dose modification by adjusting parameters such as MN design, material, and the coating film thickness. On the other hand, in coated MNs, the aforementioned parameters must be carefully chosen during product optimization. Also, due to the small size of the MN, the surface available for coating is small, which limits the amount of drug to low doses or makes it necessary to manipulate the size of the system and the number of film layers [21].

The transdermal delivery of AGM can help to omit the first-pass effect, resulting in an increase in drug bioavailability and potentially allowing for dose reduction. Using MNs will facilitate overcoming the skin barrier and make drug delivery more efficient. To the best of our knowledge, this is the first idea of delivering AGM transdermally using MN systems. The presented work is an initial step in an ongoing project aiming at the development of novel coated microneedle arrays as an alternative for oral formulations for the delivery of AGM. The further stages will comprise microneedle system development, coating, and characterization, while the aim of this study was to prepare an agomelatine-loaded gel suitable both for the transdermal drug delivery of AGM and microneedle coating. It is noteworthy that AGM, as a relatively lipophilic compound (log P = 2.83) [13], can be considered a good candidate for being successfully delivered across the *stratum corneum*. Therefore, the formulations described in this study can be presented in both aspects, as a ready-to-use formulation for dermal application and a coating material necessary to deposit the drug on the surface of microneedles. In both types of products, a detailed mechanical characterization is necessary to predict the behavior of the samples subjected to mechanical forces in the application to the skin surface or in the dip-coating process. Formulations were subjected to spectroscopic studies using Raman analysis. Also, rheological characterization and mechanical tests employing a texture analyzer were performed. Finally, two formulations were tested for potential application to the skin. For that purpose, release studies and permeation studies through human skin ex vivo were conducted.

## 2. Results and Discussion

**Figure 1:**
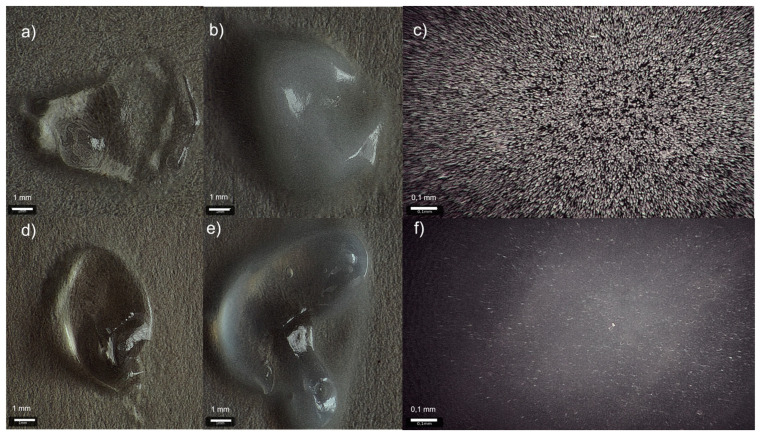
Images of the formulations obtained by the optical microscope Targano Prestige: Sus placebo (**a**), Sus AGM (**b**,**c**), Et placebo (**d**), Et AGM (**e**,**f**); objective: 10× (**a**,**b**,**d**,**e**) and 25× (**c**,**f**).

Two types of formulations were obtained and examined. One of the formulations contained agomelatine dissolved in ethanol (Et AGM gel), while in the other one, the drug was suspended (Sus AGM gel). Both placebo gels (Et placebo gel and Sus placebo gel) and the Et AGM gel were transparent, while the Sus AGM gel had a white color due to the suspended powder (Figure 1).

### 2.1. Particle Size Measurement

**Figure 2:**
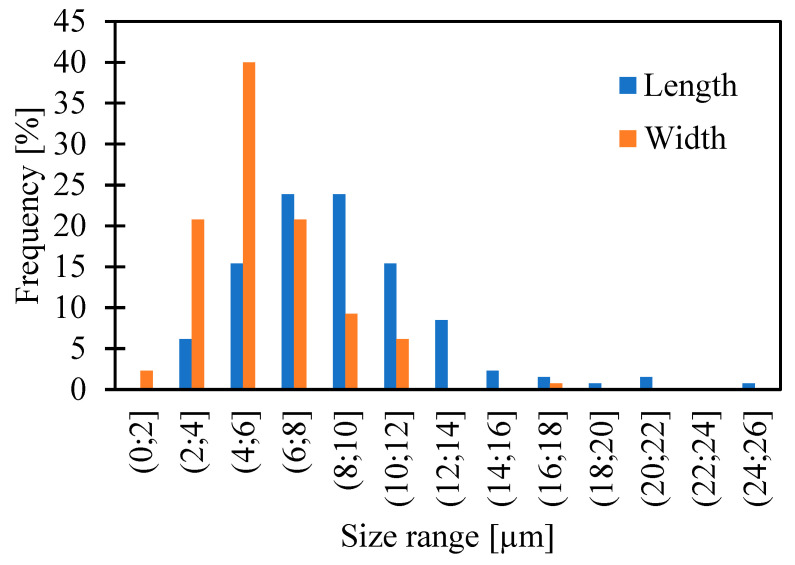
Length and width measurement results presented as the frequency of values in size ranges. “(”—range opened, “]”—range closed.

In the case of Sus AGM, a particle size measurement was performed. Figure 2 presents the size ranges for the measured values and their frequency of occurrence as a percent of all results for length or width. The minimum, maximum, median, and quartile values are shown in Table 1.

**Figure 3:**
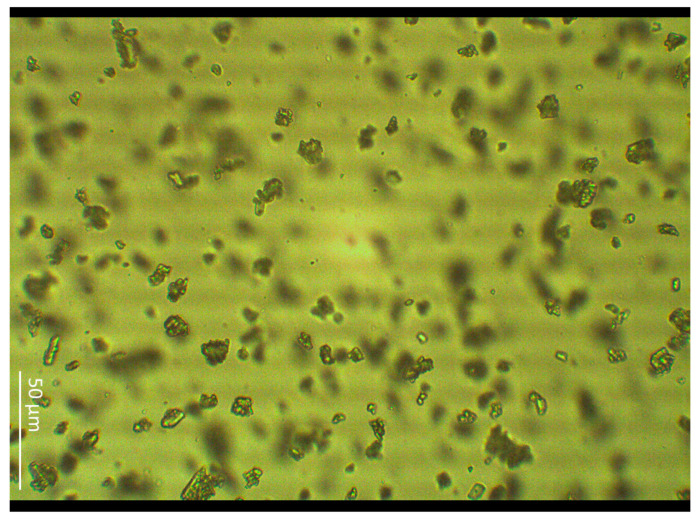
The microscopic photo of AGM crystals present in the Sus AGM gel sample. The presented image was captured at a magnification of 400×.

The shape of the particles was irregular, with both single crystals and their aggregates present in the sample (Figure 3). The particle size distribution (PSD) can influence bioavailability and the route that the drug crosses the skin [22]. Despite grinding in a mortar, which is one of the particle reduction methods [23], the crystal size reached up to 24.2 µm in length and 17.9 µm in width. Compounded ointments, made in a community pharmacy for an individual patient based on a prescription, should contain particles smaller than 90 µm [24]. The prepared Sus AGM gel met this requirement. However, the ointments prepared in community pharmacies are, in the great majority, used to cause a local effect, while the purpose of agomelatine is a systemic effect, so permeation is essential. Considering the two types of prepared gels, it can be assumed that the one containing the dissolved substance will have better permeation, and the gel with the suspended drug will have limited permeation [25].

### 2.2. Raman Analysis

**Figure 4:**
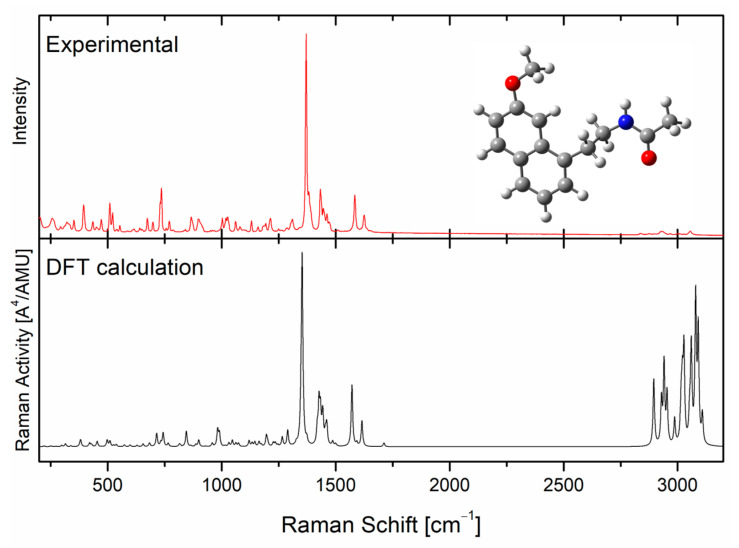
Calculation (DFT) and experimental Raman scattering spectra of agomelatine II form powders.

Figure 4 presents a comparison of the experimental results of the AGM molecule with the results of density functional theory (DFT) calculations. DFT is a quantum mechanical method that has a broad application in chemistry and physics for calculations regarding the electronic structure of molecules [26]. As can be seen from Figure 4, the AGM spectrum obtained in spectroscopic tests reflects the results obtained in the theoretical calculations well. The assignment for the intensity bands is presented in Table 2. The appearance of a band at 1030 cm^−1^ in Raman spectra is characteristic of the AGM II polymorphic form [27].

**Figure 5:**
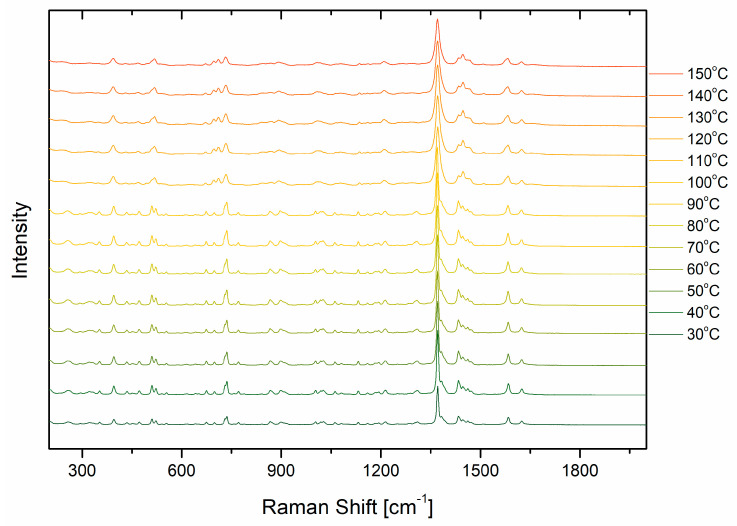
Temperature dependences of AGM powder.

**Figure 6:**
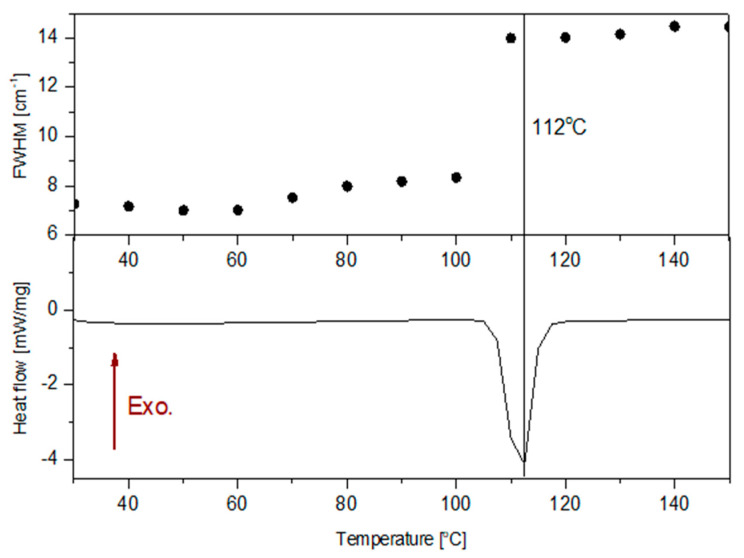
The FWHM analysis at the band at 1370 cm^−1^ (**top**). Comparison with the DSC results (**bottom**).

Further, to evaluate the stability of AGM, a temperature study was carried out (Figure 5). As seen in the figure, the changes in Raman spectra were observed at about 110 °C. Table 3 shows a comparison of the spectral parameters of the five selected bands at 30 °C and 110 °C. The analysis of the spectral parameters of the bands was performed in the Fityk 1.3.1 program [29]. Each band was fitted with a Gaussian function. The biggest changes are observed for the full width at half maximum (FWHM) and integral intensities. The change in spectral parameters confirms that a phase transition from crystalline AGM II to amorphous form occurred. Bands on Raman shifts 737, 1213, 1371, and 1584 cm^−1^ shift towards smaller wavenumbers, while the band at 1383 cm^−1^ shifts slightly towards larger wave numbers. However, any change in position up to a few cm^−1^ is within the uncertainty limits measurement; hence, these changes cannot be clearly linked to changes in the sample. As can be observed, the FWHM at 1370 cm^−1^ for AGM in the amorphous form is almost twice as large as the FWHM of the same band for agomelatine in the crystalline form. Also, instead of the three clearly separated bands between 960 and 1060 cm^−1^, there is one broad band at 1013 cm^−1^. To confirm that AGM is in the II polymorphic form, DSC studies were performed and compared with the FWHM for the Raman band at 1370 cm^−1^ (Figure 6). The melting point at 113 °C is similar to the results obtained from the Raman spectroscopy measurements. Notably, in DSC, the measurement was performed continuously, while for the Raman analysis, the temperature was raised at the same rate of degrees/min, but the temperature was maintained for the measurement time of approximately 5–8 min. However, the result for both methods is similar, approx. 110 °C.

**Figure 7:**
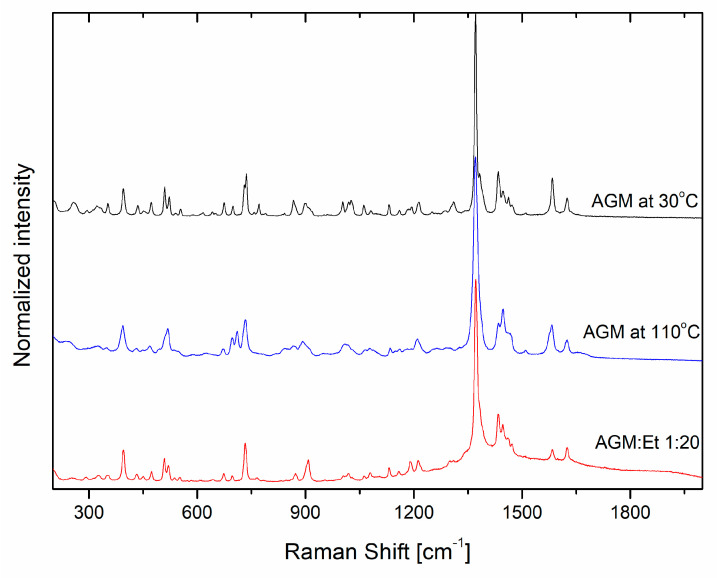
Raman spectra of AGM and AGM in ethanol solution.

After the dissolution of AGM in ethanol and solvent evaporation, some changes in the Raman spectra can be noticed with respect to polymorphic form II and the amorphous form (Figure 7). Changes in integral intensity ratio for the band at about 1584 and 1625 cm^−1^, 1061 and 1081 cm^−1^, and the broad bands in the area from 850 to 930 cm^−1^ can be observed. For the most intensive band at 1370 cm^−1^, the broadening of FWHM is observed with no clear separation of the band at 1384/91 cm^−1^. For the area between 960 and 1060 cm^−1^, three separate bands of lower intensity can still be observed.

**Figure 8:**
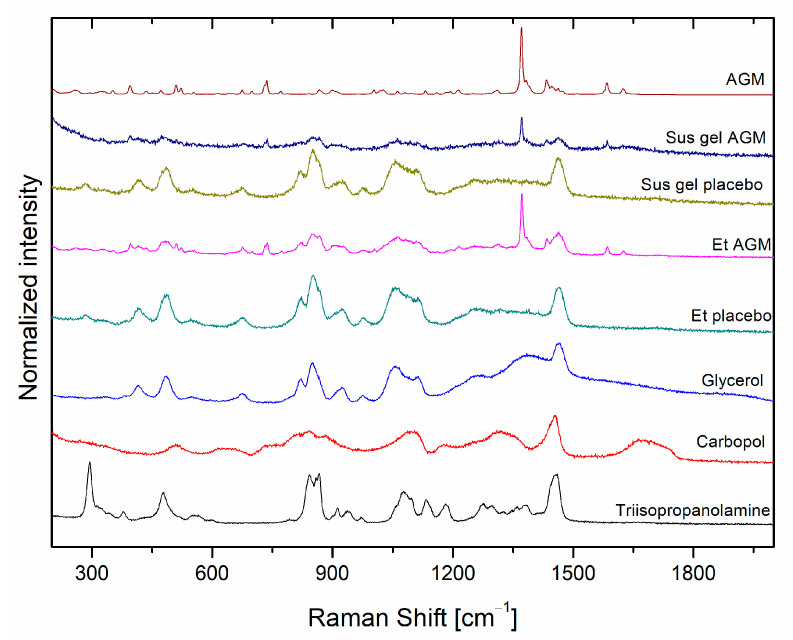
Raman spectra of AGM, components of gels (glycerol, Carbopol, triisopropanolamine), and formulations with and without AGM.

In the gel samples containing agomelatine, three characteristic peaks were observed at 737 cm^−1^, 1370 cm^−1^, and 1584 cm^−1^. According to the shape and the spectral parameters of most intensity bands at 1371 cm^−1^, AGM is of crystalline form. In all four gels, bands for the excipients including glycerol, triisopropanolamine, and Carbopol, are observed (Figure 8). However, the most intense bands come from glycerol, with the highest intensity bands at 1462 cm^−1^ (CH_2_ b), 1114 cm^−1^ (CO s), 1056 cm^−1^ (CO s), 852 cm^−1^ (CC s), 820 cm^−1^ (CC s), 486 cm^−1^ (CCO r), and 416 cm^−1^ (CCO r) [30].

### 2.3. Differential Scanning Calorimetry

**Figure 9:**
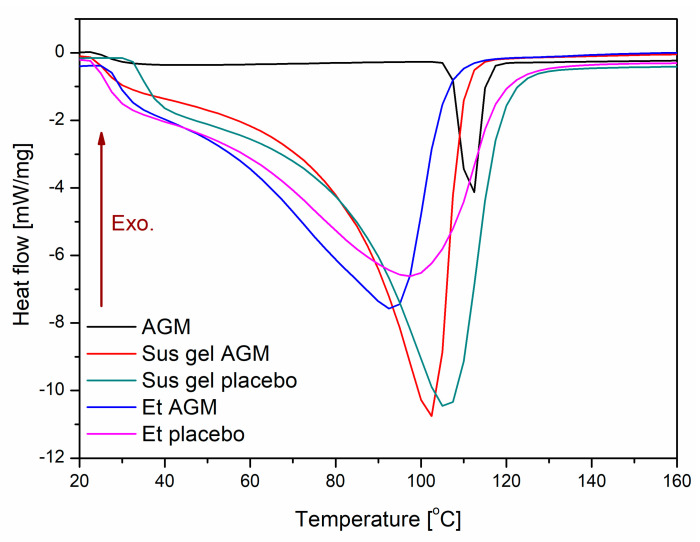
DSC thermograms of AGM II (black), Sus AGM (red), Sus placebo (green), Et AGM (blue), and Et placebo (cyan).

The DSC curves of AGM II, Sus gels, and Et gels are shown in Figure 9. The melting points at 107 °C, 104 °C, 98 °C, and 94 °C observed in the Sus placebo gel, the Sus AGM gel, the Et placebo gel, and the Et AGM gel, respectively, are different and are lower than the melting point of AGM at 112 °C. A slight decrease in the melting point can be observed in prepared formulations. The phase transition temperatures of the individual components are 46 °C for Carbopol, 92 °C for glycerol, and 76 °C and 130 °C for triisopropanolamine.

### 2.4. Loss of Volatile Components

**Figure 10:**
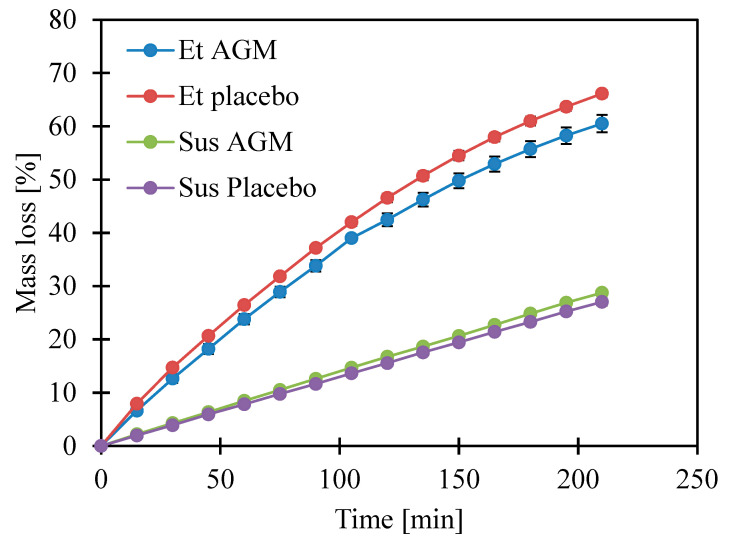
Loss of volatile components during 210 min of drying.

The drying process is important in terms of microneedle coating creation as it is one of the stages of the coating process. Moreover, it allows for the prediction of how a sample will perform during the preparations and procedure of the coating process. Taking into consideration that the gel potentially can be applied on the skin alone and compared with the microneedle-facilitated administration, a temperature of 32 ± 0.5 °C, which is skin temperature [29], was selected for this study. This temperature is also easy to obtain in the drying process of microneedle coating. As previously assumed, the volatile loss observed for the ethanol gels was faster. Also, for these gels, the graph ran with a visible inflection, while for Sus AGM and Sus Placebo, linearity was observed (Figure 10). There was no significant difference in the group of suspensions after 210 min of the test, while in the group of ethanol gels, the gel without the active substance showed a greater loss of volatile components.

### 2.5. Texture Profile Analysis and Spreadability Study

**Figure 11:**
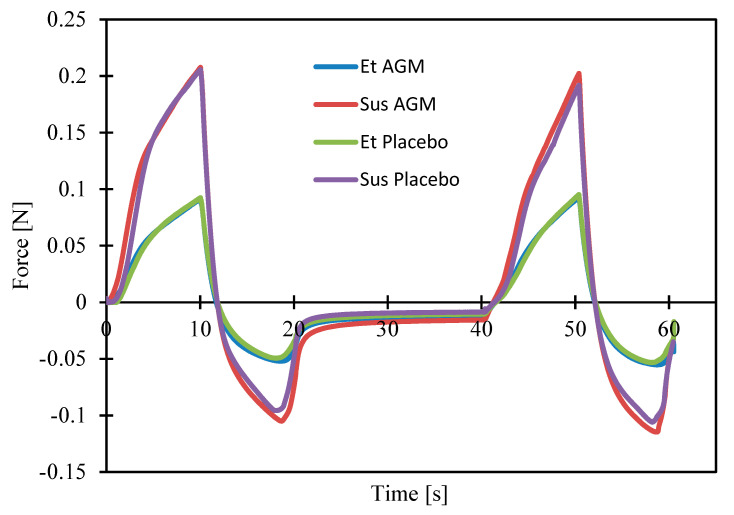
Texture profiles of placebo gels and gels loaded with agomelatine.

**Figure 12:**
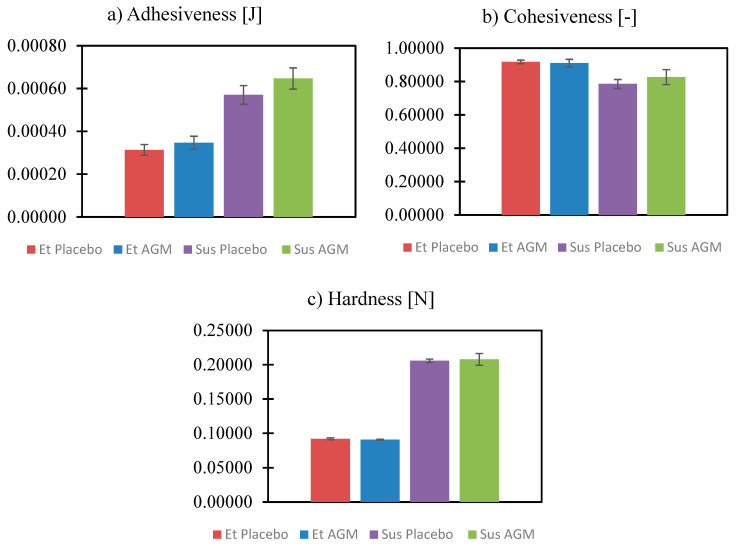
(**a**–**c**) Comparison of the values from texture profile analysis for the ethanol gel and suspension, both with the placebo and loaded with agomelatine. Adhesiveness is shown as an absolute value.

**Figure 13:**
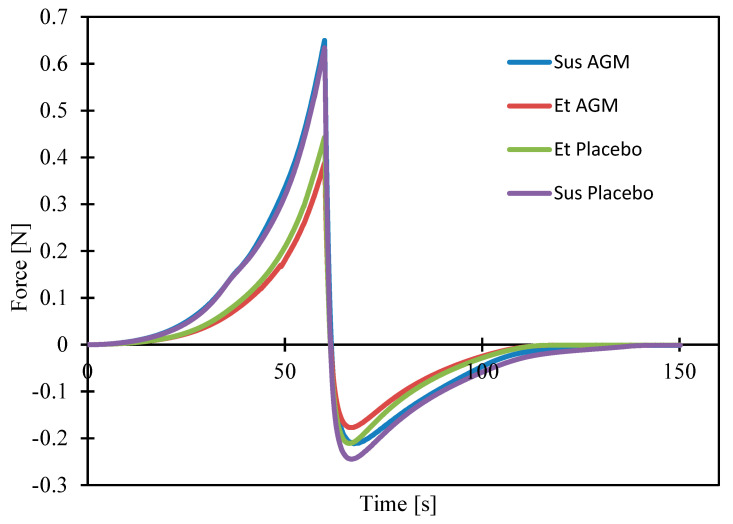
Spreadability study of placebo gels and gels loaded with agomelatine.

**Figure 14:**
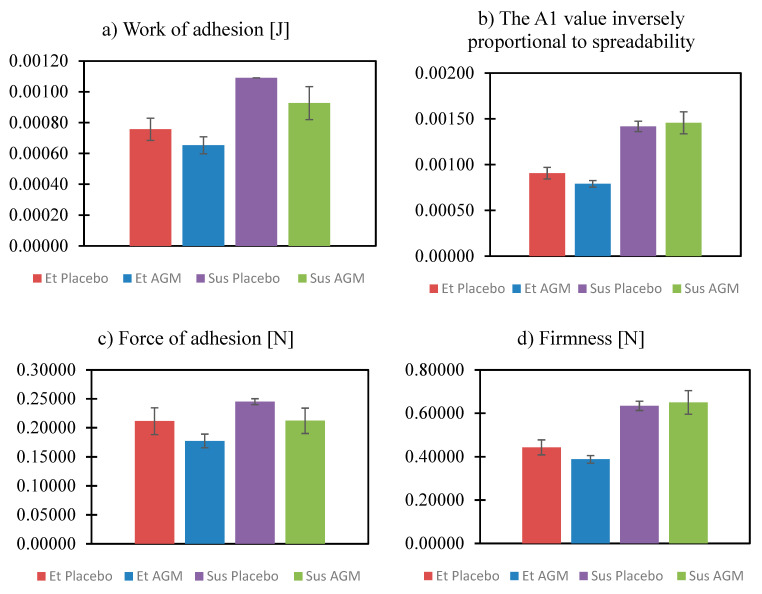
(**a**–**d**) Comparison of the results from the spreadability study for the ethanol gel and suspension, both with the placebo and loaded with agomelatine. Work of adhesion and force of adhesion are shown as absolute values.

Texture analysis was performed to compare the two formulations and identify if the presence of AGM modifies their mechanical properties. Investigating parameters such as cohesiveness, adhesiveness, etc., is essential for the effective coating process. Moreover, the spreadability study helped to assess which formulation could be more suitable in the case of topical application. Figure 11 and Figure 12a–c present the results of the texture profile analysis for the tested samples, and Figure 13 and Figure 14a–d present the outcome of the spreadability test, with the A1 value calculated as the work of penetration during the sample compression, which is inversely proportional to spreadability.

The presence of AGM does not affect parameters such as adhesiveness, cohesiveness, hardness, the value of the work of adhesion, firmness, the force of adhesion, or spreadability in the group of ethanol gels or in the group of suspensions. Suspensions exhibit greater adhesiveness, hardness, value of the work of adhesion, and firmness, while ethanol gels are characterized by greater cohesiveness and spreadability. There was no significant effect of the presence of AGM or the type of gel on the force of adhesion.

### 2.6. Rheological Studies

**Figure 15:**
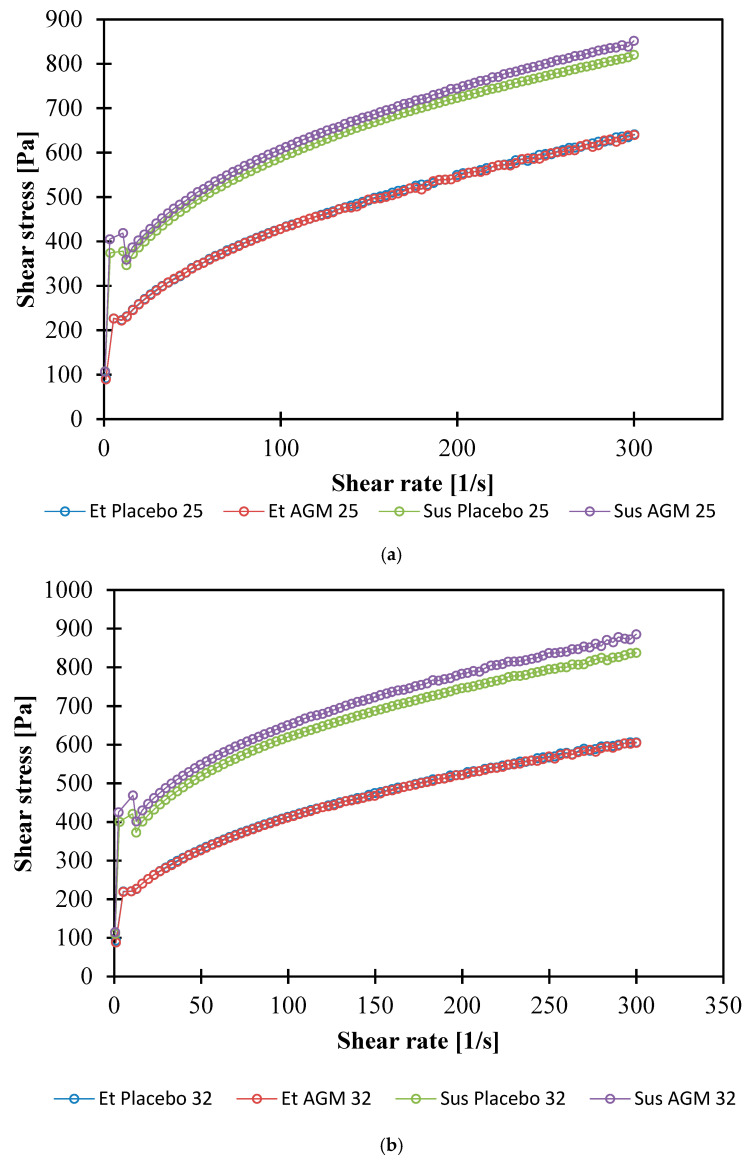
(**a**,**b**) Flow curves for the placebo and agomelatine-loaded formulations obtained at 25 °C (**a**) and 32 °C (**b**).

Figure 15a,b show the flow curves determined at 25 °C and 32 °C. The curves were fit to the Herschel–Bulkley model:

(1) $$\tau =\tau_{0}+K\;{\dot{\gamma }}^{n}$$

where τ—shear stress [Pa], τ_0_—yield point [Pa], K—consistency index [Ns^n^/m^2^], $\dot{\gamma }$—shear rate [1/s], and n—flow index [-]. The values of the n and K coefficients are presented in Table 4, and the yield stress values are shown in Table 5.

The Herschel–Bulkley (HB) model is a model of non-Newtonian fluid, which means that the correlation between the shear rate and the shear stress is non-linear. The presence of this phenomenon differs from the HB model from the Bingham model [31]. Also, there is a yield point that can be understood as the amount of shear stress a formulation can absorb before it begins to flow. A value of the flow index (n) lower than 1 is characteristic of shear-thinning behavior. All investigated formulations showed a flow index lower than 1. The consistency index is dependent on the flow index and is usually presented along with the n factor [32]. It should be emphasized that the wall slip effect was not investigated in this study. This complex phenomenon can be observed in various concentrations and types of hydrogels, including Carbopol-based formulations [33]. The parameters of this experiment were selected so that the wall slip effect was minimized and did not disturb the flow curve.

There was no significant effect of AGM on the K and n parameters or the yield point at both temperatures. The K parameter for suspensions is higher than for ethanol gels, while the n parameter is lower. The mentioned parameters for ethanol gels do not differ significantly with temperature. However, in the case of suspensions, the temperature affects the values of the coefficients. The K coefficient is higher, and the n parameter is lower at 32 °C.

The yield point for the suspensions is higher than for the ethanol gels at both temperatures. In the ethanol gels, the yield point turned out to be independent of temperature, while the suspensions showed a lower yield point at higher temperatures.

**Figure 16:**
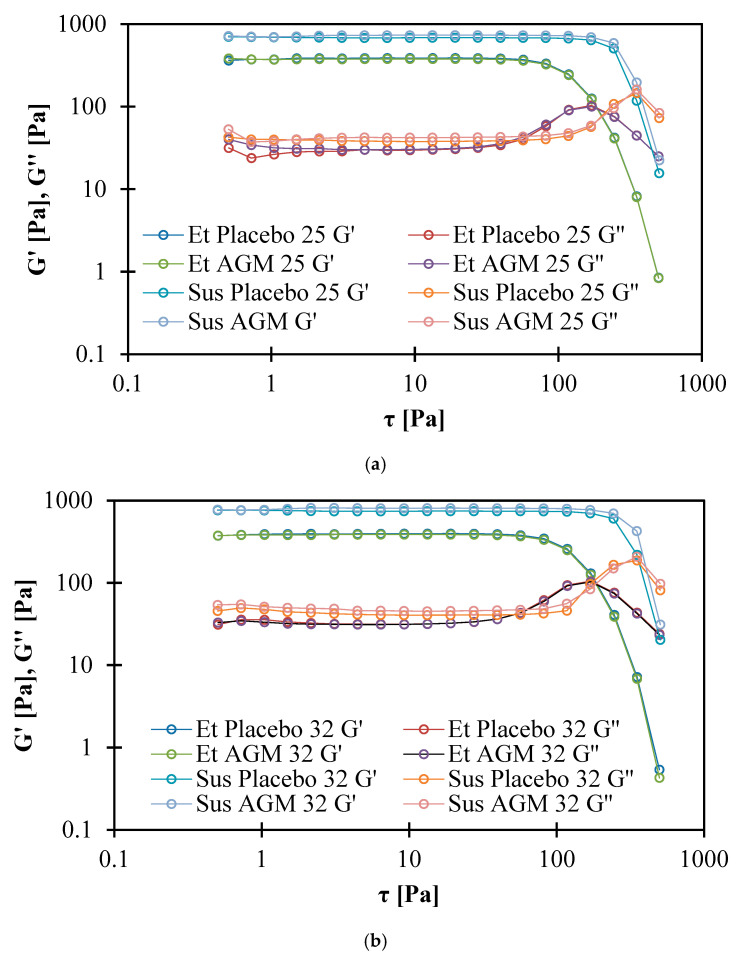
(**a**,**b**) Cross point of storage modulus G′ and loss modulus G″ at 25 °C (**a**) and 32 °C (**b**).

**Figure 17:**
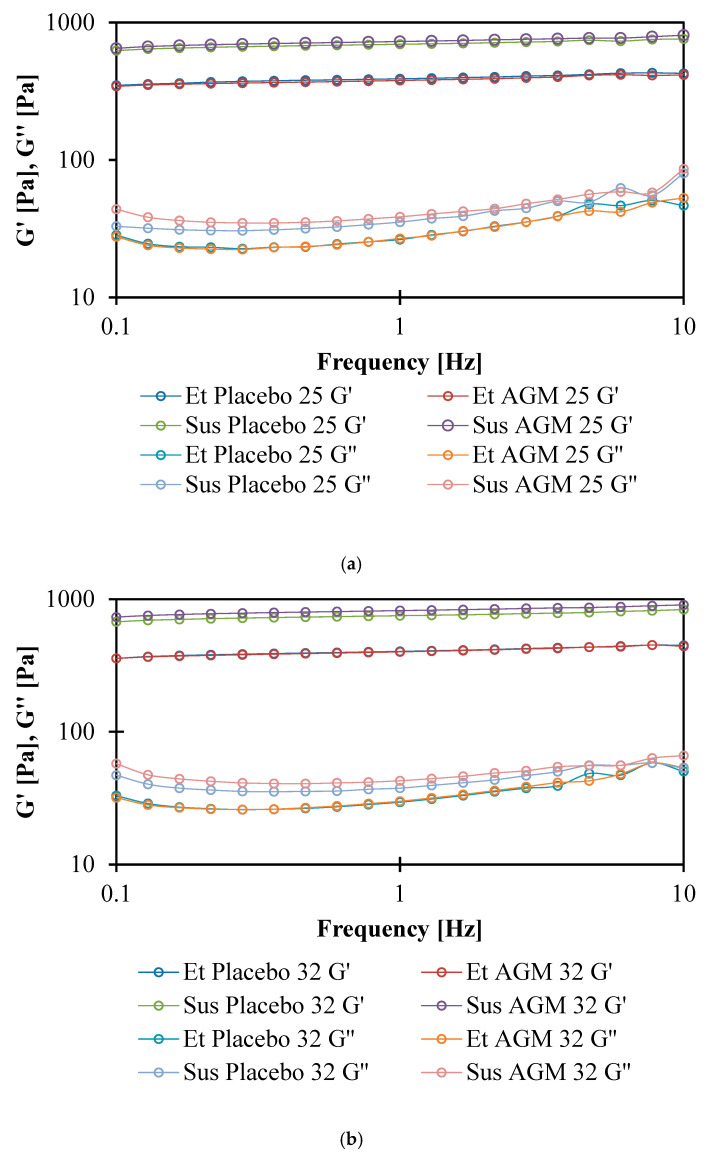
(**a**,**b**) Storage modulus G′ and loss modulus G″ in a variable frequency of oscillation at 25 °C (**a**) and 32 °C (**b**).

The numerical values for the module intersections are presented in Table 6, and the graph is presented in Figure 16a,b. AGM addition does not affect the τ value of the cross point of the ethanol gels, while AGM suspensions have higher τ values. At both temperatures, the suspensions have higher τ values than the ethanol gels. Increasing the temperature does not significantly affect the τ values for ethanol, while the suspensions show higher values at higher temperatures. The G′ modulus is higher than G″ for all gels, and both are slightly dependent on frequency, which suggests a weak gel behavior (Figure 17a,b) [34].

### 2.7. Release Studies

**Figure 18:**
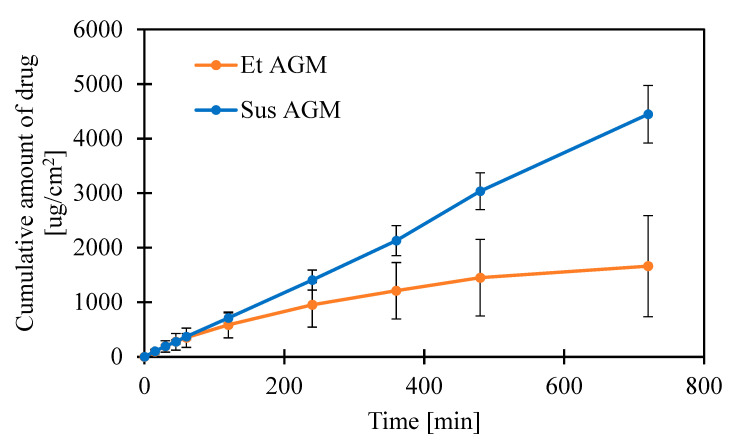
Cumulative amount of drug released through the membrane per surface area [µg/cm^2^].

**Figure 19:**
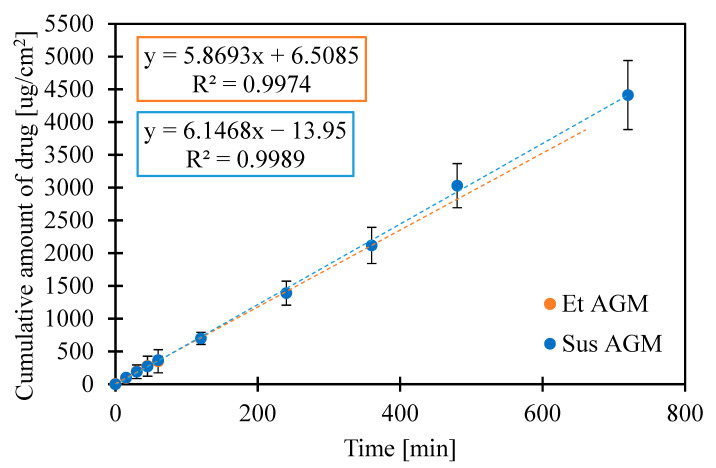
Comparison of the linear parts of the graphs. Up to 720 min for Sus AGM and a part up to 60 min for Et AGM (extrapolated for better visibility) [µg/cm^2^].

Figure 18 shows the result of the release study using a cellulose membrane. The addition of ethanol to the acceptor fluid was dictated by the low solubility of AGM and the need to maintain sink conditions. A similar approach was presented by Ahmed et al. [35], where for their release study, a phosphate buffer and ethanol mixture in the proportion of 7:3 was employed, and by Dyja et al. [36], where phosphate-buffered saline at pH 6.8 containing 40% (*v*/*v*) ethanol was used. The slope was determined from the linear part of the graph (Figure 19). It can be concluded that AGM from the Sus gel formulation is released more efficiently than from the Et AGM gel. The amount of drug released at the end of the study is higher for Sus AGM (Table 7). However, the slope of the linear part of the graph is similar in both cases. For the Et AGM gel, a two-stage release profile is observed.

### 2.8. Ex Vivo Permeation Studies on Human Cadaver Skin

**Figure 20:**
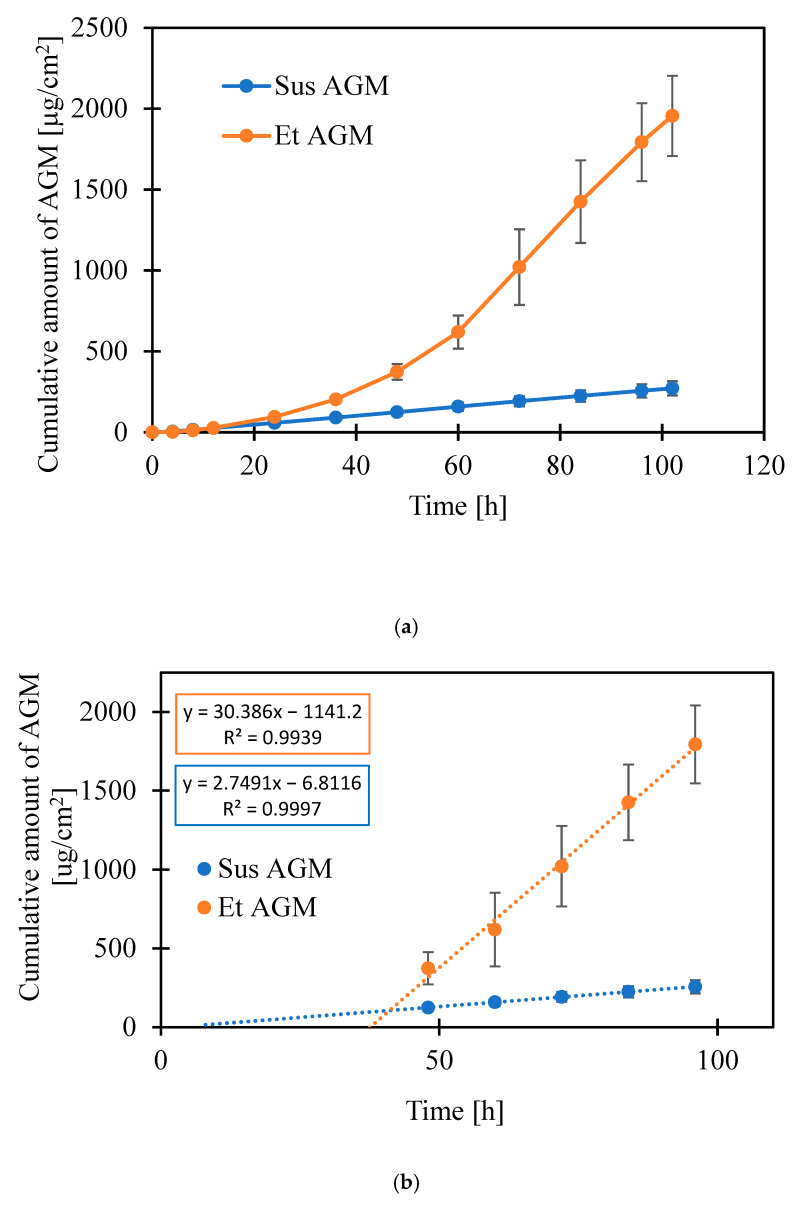
(**a**,**b**) Cumulative amount of drug permeated through the skin per surface area [µg/cm^2^].

Permeation on human skin was also performed to compare both formulations. Various artificial membranes could be used as a barrier in a permeation study, but human skin is still a golden standard for this purpose. A steady state flux (Jss) was determined as the slope of the linear part of the graphs, and the lag time was calculated as the intersection of the linear part of the graph with the horizontal axis (Table 8). As shown in Figure 20a,b, the drug permeated from Et AGM gel through the skin more effectively, but the linear part of the graph for the Et AGM gel occurred later than for the Sus AGM gel. It must be emphasized that the timeframe in the ex vivo tests was extended in comparison to the drug release tests, as the permeation rate was generally slower. The observed effect is quite obvious, taking into consideration the biological barriers and the thickness of the human cadaver skin compared to the regenerated cellulose applied in the drug release tests.

**Figure 21:**
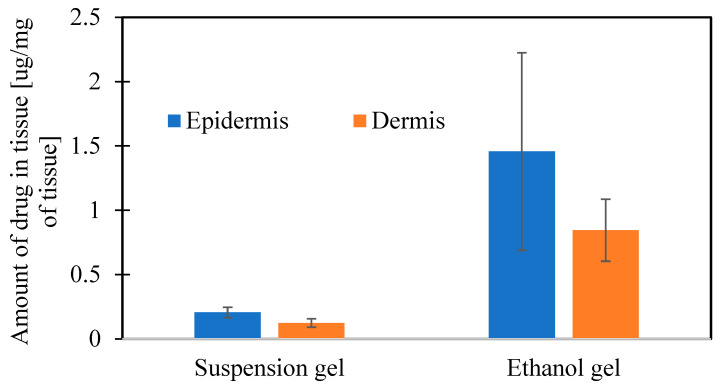
Amount of drug retained in different layers of the skin.

The analysis of skin layers after the study showed that a higher amount of drug from the Et AGM gel was entrapped in the skin. The amount of drug in both investigated layers, i.e., the epidermis and dermis, was higher for Et AGM. There was no difference in the amount of drug between the dermis and epidermis in the Et AGM gel, while in the Sus AGM gel, more drug was accumulated in the epidermis (Figure 21).

In summary, coated MNs consist of solid bases of various sizes and shapes, covered with a coating material, which makes the approach very flexible. The delivery of various substances can be provided by one type of solid system just by changing the coating. Moreover, the mechanical properties of the MNs usually remain unaffected by the coating process. The idea of coated microneedles is as follows: the MN system is inserted into the skin; then, the interstitial fluid present in the tissue starts to interact with the coating. The excipients forming the coating material are usually water-soluble, which facilitates detaching the coating from MNs. This process can last seconds or minutes but should end before the MN system is removed from the skin [37].

There are various methods of coating MNs. Spray-coating uses a nozzle to cover an MN with small droplets of a coating solution. Then, the material undergoes coalescence and forms a film on the surface of the MN. Piezoelectric inkjet printing employs a piezoelectric crystal and an electric field to produce coating solution drops. Gas-jet drying includes applying the material and drying at an angle [38]. Some approaches include more sophisticated systems for coating MNs using brush-like sheets [39] or a roller [40]. Among the coating methods, dip-coating is the most simple one while still being effective, low-cost, and easy to modify [41].

The optimized dip-coating process should be characterized by a homogeneous and repeatable coating. MNs should be covered with a coating material, but the base of the array should remain clear, and the spaces between MNs should be preserved. Besides the process parameters, such as the distance at which the MNs are immersed, withdrawal speed, or time of drying between the dips, the properties of the coating material are crucial [37]. Inaccurate material properties could result in the flowing of the solution during drying or suction of the solution between the microneedles by capillary forces.

Carbopol^®^ is an acrylic acid copolymer that can form a gel in concentrations of 0.5–1.5%. It was selected for the formulations because of its stability and non-irritating effect on the skin [42]. There are several types of Carbopol^®^, which differ slightly in terms of their properties. In this study, Carbopol^®^ EZ-3 was employed. It has thickening and suspension-stabilizing properties and is used to create transparent and rigid gels. All these features are desirable for forming a good coating material. The greater viscosity of a coating solution results in a thicker film layer on MNs, which facilitates the increase in drug loading [37]. After dispensing the polymer in water, it swells intensively, but a neutralization step is needed to achieve a gel-like consistency. The neutralization causes the ionization of carboxylic acid groups along the polymeric chain. This favors the cross-linking process [43]. However, in the presence of ethanol, there is a risk of Carbopol salt precipitation. To avoid such risk, triisopropanolamine (TIPA) was chosen as a neutralizer. TIPA is an effective neutralizing agent even in hydroalcoholic systems of up to 90% alcohol [44]. The addition of ethanol to one type of gel was dictated by AGM’s low water solubility. Moreover, ethanol also possesses skin permeation enhancement properties for polar and non-polar molecules, which can facilitate AGM delivery [45]. Preparing two types of formulations, i.e., one with AGM dissolved in ethanol and one with AGM suspended in a gel, creates an opportunity to investigate the differences in terms of mechanical, rheological, and permeation properties. Also, these two types of formulations are discussed below as simple potential transdermal delivery formulations and materials for coating MNs.

Raman spectroscopy is a widely used, non-destructive technique to determine the sample chemical structure, polymorphism, and possible interactions such as molecular interactions, the molecular environment, or traces of other dopants. It allows for the identification and confirmation of the presence of an active pharmaceutical ingredient (API) in a sample. As a result of Raman analysis, the presence of AGM in both formulations was confirmed, and all ingredients were identified in the spectra. However, it should be mentioned that the freeze-drying process needed to be included. This step was necessary because testing the transparent sample distorted the results due to interference caused by the received Raman signal from the substrate on which the material (mica or quartz) was tested. Also, determining the polymorphic form of AGM after ethanol evaporation presented problems because the analysis results were ambiguous. The AGM band, after dissolution in ethanol and evaporation of the solvent, does not clearly indicate the AGM form, but rather, it shows characteristics of the amorphous band and the crystalline form II at the same time. From the pharmaceutical point of view, usually, the amorphous form of an API is more desired due to the better dissolution rate compared to the crystalline form of poorly water-soluble drugs. However, the amorphous form tends to recrystallize because it is in the higher-energy state, which makes it thermodynamically unstable [46]. AGM as a powder was found to be in an II polymorphic form and stable up to 110 °C, which is essential during further processing. The dip-coating method could be optimized in a simple manner without drastic process conditions, such as heating above 110 °C. Out of the six polymorphic forms of AGM, only forms I and II are suitable for industry processing, while the II form is the most thermodynamically stable [47] and shows good stability during heating [48,49,50].

The observations from the drying of both gels allow for the conclusion that the Et AGM gel will dry faster, which may benefit the dip-coating process. The time of 210 min is enough to see the slight tendency of the Et gels to stabilize their mass, while it is still too short to see the Sus gels stabilize. It is quite obvious that the difference is because of the presence of ethanol in Et gel samples. Faster drying will enable a more rapid application of subsequent layers of material, accelerate the process, and prevent the liquid from flowing down the microneedle. This is especially important when no additional step of drying is involved; e.g., Gill et al. [51] applied a 24 h air-drying time for MNs coated with deionized water-based and organic solvent-based solutions.

Texture profile analysis and spreadability tests are widely used in studies of semi-solids. They help gain insight into how a product will behave when extracted from a container during application or handling [52]. Hardness reflects the force needed to cause a deformation of a gel. A lower hardness value facilitates easier gel application on a site [53]. Adhesiveness shows the force needed to overcome the attractive force between the sample and the probe [52]. In contrast, cohesiveness is the tendency of a gel to hold together. Spreadability is considered important in the case of the application of a gel straight on the skin surface. It guarantees the ease of handling and the comfort of use. The procedure performed during the texture profile analysis, to some extent, is similar to the dip-coating process because the probe dips in a vessel filled with a gel. The Sus AGM gel showed better adhesiveness to the probe, which can suggest that it will hold to the MN and not fall off during the process or slip onto the base of the array while drying. The Sus AGM gel presented higher hardness and firmness, which made it harder to spread on the skin. Importantly, the addition of the drug did not influence the properties of the coating material. The lower hardness and higher cohesiveness of the Et AGM gel can lead to the conclusion that it would perform better than Sus AGM as a gel for topical application. Additionally, considering the spreadability properties, the Et AGM gel was also superior. In this case, the addition of the drug also did not affect the properties of the formulation. However, in the case of the multiple-layer coating, the addition of ethanol in the Et AGM gel may facilitate the uniformity of layers due to the wetting effect and reduced surface tension [54].

The shear-thinning properties and correlation with the HB model of both gels are probably due to the use of the Carbopol^®^ polymer, which is in agreement with the literature. In a previous study, the values of the HB model coefficients for the same concentration of polymer were K = 50.102 [Pa s^n^], τ_0_ = 153.33 [Pa], and n = 0.3846 [43]. However, the pH value, method of preparation, and overall additional ingredients influenced the final values of the investigated parameters. Glycerol was found to influence hydrogen bonding between water, a solvent, and a polymer, which further interferes with the swelling and viscoelastic properties of polymers [55]. Fresno Contreras et al. [56] showed that ethanol/water hydroalcoholic gels based on Carbopol^®^ Ultrez 10 also present pseudoplastic and shear-thinning behavior; however, in that case, the amount of ethanol was lower (15%) compared to the ethanol gels presented in this study. The yield point of the Sus gels is higher than that of the Et gels at both temperatures. This is in agreement with the TPA study, which showed that the Sus gels have greater hardness. This conclusion is also consistent with the result of the amplitude sweep test. The points of intersection for the storage and loss moduli for the Sus gels were higher, which means that higher shear stress is needed for viscous properties to overcome elastic properties. The decrease in the yield point for Sus AGM with the increase in temperature could be explained by the temporary reduction in stability between polymer chains [43]; however, the change in temperature did not significantly affect the Et gels. Rheological properties are important in terms of a subjective feeling during topical product application. Previously investigated marketed topical gel products (Voltaren 1%, Voltaren Max 2.3%, Nitrocard 20 mg) also presented non-Newtonian pseudoplastic behavior, and the flow curves were fitted to the HB model [57]. The yield point for these products investigated at 20 °C is in a wide range—from 26.11 to 129.8 [Pa]. Et AGM’s yield point also stays within that range, while the yield point for Sus AGM is almost twice as high. However, a 5 °C difference in the measurement temperature should be emphasized, as the coefficients change with temperature. Moreover, the materials suitable for the dip-coating process usually contain polymer material and are of non-Newtonian behavior. This is explained by the fact that a low viscosity is required under a higher shear rate during the coating process, and higher viscosity facilitating good adhesion of the coating solution to the material is required under a lower shear rate, e.g., during drying [58]. This leads to the conclusion that the investigated gels could be used both for topical and MN coating purposes when rheological properties are considered.

Considering the results of previous mechanical studies, it was concluded that both formulations can be potentially applied to the skin. Despite the very simple composition of the gels, permeation studies were performed to investigate the potential ability of both formulations to cross the skin barrier without microneedle assistance. The Sus AGM gels present a linear correlation between the cumulative amount of drug released and time, while the graph for the Et AGM gels shows two-stage release. The first phase, up to 1 h, is almost linear, while in the second phase, the graph flattens. The amount of drug released at the end of the study (per cm^2^) is higher for Sus AGM. This result is in conflict with an expectation that the drug would permeate more efficiently from a formulation where it is dissolved. A potential explanation for this phenomenon can be as follows. In the Sus AGM gel, the drug was suspended in the matrix, so it is possible that some of the particles were sedimented, and thus, the contact substance membrane was present during the whole study. However, the Et AGM gel included a drug in a dissolved form. The bottom layers of the gel were in contact with the membrane, while the upper layers were not. Probably, during the initial faster release, the amount of substance from the lower layers of the gel was easily available to permeate. As the study ran, the reservoir in these layers was probably exhausted, while the drug from the higher layers of the gel diffused into the lower layers slowly due to the higher viscosity of the medium. This could have caused the impression of dose exhaustion. Comparing the results from the release and permeation studies, it can be concluded that the results are opposite to each other. In the ex vivo permeation study, the Et AGM gel permeated more efficiently, and the amount permeated from this formulation was significantly higher compared to the Sus AGM gel. However, the linear correlation between the amount of drug permeated per cm^2^ and time was achieved much later in the case of the Et AGM gel. The difference in permeation may be caused by the presence of ethanol in the Et AGM formulation and its ability to act as a permeation enhancer [45]. This effect was not seen in the previous study on cellulose membranes, which proves the importance of testing the formulation on human skin. Moreover, the AGM from the ethanol gel permeated slower at the beginning and faster in the second phase of the study. This may be due to the prolonged effect of ethanol on the skin. Such an effect was not observed for Sus AGM, which does not contain ethanol. In the study conducted by Said et al. [12], the permeation of different AGM-loaded microemulsions (ME) was evaluated. The permeation from the tested ME formulations ranged from 0.6 to 5.0 mg/cm^2^ after 24 h, while in another study conducted by Ahmed et al. [15], the permeation of AGM from a hydrogel with a nanostructured lipid carrier was 89.440 ± 2.586 µg/cm^2^. For the Et AGM gel, it was 0.094 mg/cm^2^ after 24 h. However, the study parameters, such as skin origin or acceptor medium composition, varied in these three studies, so the results cannot be compared. The amount of AGM present in the skin was higher for the Et AGM gel, while for the Sus gel, the epidermis contained more AGM as the layer that directly contacts the formulation. The presence of the higher amount of AGM in the Et AGM gel skin sample is probably due to the ethanol action on the skin, which includes an enhancement in drug solubility in *stratum corneum* lipids, changes in skin hydration, extraction of lipids or increases in their fluidity, and alterations in keratinized proteins [59].

## 3. Materials and Methods

### 3.1. Materials

Agomelatine was purchased from Jiangxi Synergy, Yichun, Jiangxi, China. Carbopol^®^ EZ-3 polymer was obtained from the Lubrizol Corporation, Brussels, Belgium. Glycerol 85% was obtained from Fagron, Olomouc, Czech Republic. Ethanol 96%, ethanol 99.8%, and potassium dihydrogen phosphate were purchased from POCH, Gliwice, Poland. Deionized water was obtained from Simplicity^®^ Water Purification System, (Merck Millipore, Burlington, MA, USA). Orthophosphoric acid 85% was purchased from VWR International S.A.S, Briare, France. Sodium azide, phosphate-buffered saline (PBS), triizopropanolamine 95%, and acetonitrile were purchased from Sigma-Aldrich, St. Louis, MO, USA. Statistical analyses were performed using Statistica software version 14.1.0.4, Cloud Software Group, Inc. (Fort Lauderdale, FL, USA); TIBCO software (https://www.tibco.com/) (Palo Alto, CA, USA) (2023).

### 3.2. Preparation of Gels

Two different gel formulations were prepared, including one with AGM previously dissolved in ethanol (Et AGM) and another with AGM suspended in the finished gel (Sus AGM). The gels were prepared in plastic containers with caps to avoid evaporation. Glycerol and water were weighed into the container, and then Carbopol^®^ EZ-3 was spilled on the surface to allow the wetting of the polymer. The mixture was stirred with a mechanical stirrer CAT R-50 for half an hour at 600 rpm. AGM dissolved in ethanol was added 5 min before the end of mixing. In order to obtain a gel, the previously prepared 40% triisopropanolamine solution was added and mixed carefully using a glass stirring rod. The pH value of the gels ranged from 7.0 to 7.3, which means that the polymer was neutralized. Suspension samples were prepared analogously, but without adding ethanol, and AGM was suspended in the finished gel by grinding in a mortar. Gels containing the active substance and a placebo (Et Placebo, Sus Placebo) were prepared for further studies. The composition of the gels is shown in Table 9. Gels with a similar composition were used in previous work [60].

### 3.3. Particle Size Measurement

For the Sus AGM gel, the particle size was measured. An amount of 100 µL of the gel sample was placed on the glass slide and covered with a coverslip by applying soft pressure [61]. A square window of 2 × 2 mm was marked in the central part of each coverslip. Within this window, a photo of a 250 × 200 µm area was captured by a B3 Professional Series (Motic, Xiamen, China) optical microscope equipped with Digital Moticam 2300 (Motic, Xiamen, China). The length and width of each crystal visible in this area were measured using Motic Images Plus 2.0 software (Motic China Group Co., Ltd., Xiamen, China). The measurement was performed for three different samples.

### 3.4. Raman Analysis

Nonpolarized Raman scattering spectra were measured by a Renishaw inVia micro-Raman spectrometer (Wotton-under-Edge, Gloucestershire, UK) in the backscattering geometry. All spectra were recorded with an excitation wavelength of 785 nm using a solid-state laser as the incident radiation of the light, with a power of less than 1 mW. The spectral resolution was 2 cm^−1^. The light from the laser was focused on a sample with a 50× LWD (Long Working Distance) microscope objective, NA = 0.5. The Raman scattering spectra of the AGM powder and gels were investigated in the spectral range of 100–3200 cm^−1^. Temperature measurements were performed using a Linkam THMS 600 temperature stage, which was connected to a TMS 94 temperature controller. The latter exhibited an accuracy of ±0.1 °C. The micro-Raman spectroscopy study was conducted within a temperature range of 25 to 150 °C. To determine the effect of the ethanol on the Raman spectrum, AGM was dissolved in ethanol at a ratio of 1:20. In order to enable Raman analysis of the four formulations, the gel samples were subjected to a freeze-drying process under conditions (time: 66 h, freezing temp.: −35 °C, drying temp.: −40 °C) using an Epsilon 2-4 LSCplus Martin Christ lyophilizer (Martin Christ, Osterode am Harz, Germany).

In order to interpret the experimental results from Raman scattering, in the Gaussian 09 program [62], quantum chemical calculations using density functional theory (DFT) methods with Becke’s three-parameter exchange combined with the Lee–Yang–Parr correlational functional (B3LYP) and the standard 6-31G(d,p) as a basic set, were performed. GaussView 5.0.9 software was utilized to propose the initial geometry of the investigated molecules and to visually inspect the normal modes. The molecule of AGM II for the calculation of the Raman spectrum was taken from the Cambridge Crystallographic Data Centre website [63].

### 3.5. Differential Scanning Calorimetry

Differential scanning calorimetry (DSC) was performed on a Netzsch DSC 200 calorimeter (Netzsch, Selb, Germany). The samples, with no additional preparation, were heated to 200 °C at a rate of 10 °C/min and cooled to 20 °C at the same rate in an argon atmosphere; the sample was in an aluminum pan. There was one heating and cooling run (cooling run not shown). As a reference, an empty aluminum pan was used.

### 3.6. Loss of Volatile Components

Taking into consideration a relevant amount of volatile components, the loss of these was studied in controlled conditions. A weighed amount of each gel was applied to three plates (diameter: 70 mm, depth: 2.5 mm) of known weight and dried for 210 min at 32 ± 0.5 °C in a dryer (Binder ED 115, BINDER GmbH, Tuttlingen, Germany). The plates were weighed at intervals of 15 min. The statistical analysis was performed using Statistica software, including an ANOVA test, *p* = 0.05.

### 3.7. Texture Profile Analysis and Spreadability

The measurement was performed using a Shimadzu AGS-X texture analyzer (Shimadzu, Kyoto, Japan), and TrapeziumX 1.52 software (Shimadzu, Japan) was employed for calculations. This study was conducted with two methods, enabling the determination of different parameters: hardness, adhesiveness, cohesiveness, firmness, spreadability, and force of adhesion. Each formulation was examined in triplicate, with a fresh sample portion used in each replicate test. All measurements were performed at ambient temperature. The statistical analysis was performed using Statistica software, including an ANOVA test, *p* = 0.05.

#### 3.7.1. Texture Profile

A 20 mL sample of the gel was placed in a 25 mL glass beaker situated in a holder. A cylindrical sensor, 10 mm diameter, was positioned just above the surface of the sample. During the measurement, the sensor moved at a speed of 1 mm/s and was immersed in the sample twice to a depth of 10 mm, with a 20 s interval between dips.

Hardness was defined as the maximum force value recorded during the first compression cycle, cohesiveness as the quotient of the areas of the peaks corresponding to the second and first compression cycles, and adhesiveness as the work of the probe withdrawal corresponding to the area of the negative peak recorded between the first and second cycles of compression.

#### 3.7.2. Spreadability

The sample was placed in an acrylic conical (angle: 90°) container, with a diameter of 30 mm, and secured with the bottom fixture. A sensor, with a conical shape to match the shape of the container, was positioned just above the surface of the sample. During the test, the sensor was dipped into the gel at a speed of 10 mm/min to a depth of 10 mm.

Firmness and adhesion strength were determined as positive peak maximum and negative peak minimum, respectively. Spreadability and adhesiveness were presented as the work of sample compression and probe withdrawal, respectively.

### 3.8. Rheological Measurements

Rheological measurements were performed using a HAAKE RheoStress1 rheometer (Thermo Fisher Scientific, Waltham, MA, USA) with a titanium parallel plate system (diameter: 35 mm) and HAAKE RheoWin Job Manager and Data Manager software. The statistical analysis was performed using Statistica software, including an ANOVA test, *p* = 0.05.

The gel sample was applied onto the lower plate. The upper plate was set in position with a 1 mm gap to the upper plate. A thermostat Thermo Haake^®^ DC 30 was used to provide stable temperature conditions, and all formulations were tested at both 25 ± 0.2 °C and 32 ± 0.2 °C. Four types of measurements were performed. Each formulation was examined in triplicate, with a fresh portion of the sample used in each test. The following measurements were performed:

1. Measurement of the shear stress at a shear rate of 0–300 [1/s].
2. Measurement of deformation depending on the shear stress. Determination of the yield point by defining the cross point of two curves.
3. Oscillatory measurement: Amplitude sweep. Determination of the cross point of storage modulus G′ and loss modulus G″ and sections of elastoviscosity at 1 Hz frequency.
4. Oscillatory measurement: Frequency sweep. Measurement of storage modulus G′ and loss modulus G″ in a variable frequency of oscillation.

### 3.9. Release Studies

Release studies were conducted using Franz Diffusion cells (Teledyne Hanson Research, Chatsworth, CA, USA) with a 1 cm^2^ orifice and a cellulose membrane (SnakeSkin^TM^ Dialysis Tubing, 10K MWCO, ThermoFisher Scientific, Waltham, MA, USA). About 0.5 g of each formulation was applied on the surface of the membrane, and the donor chambers were covered with parafilm to prevent the gel sample from drying out. The temperature was set at 32 ± 0.5 °C, with constant stirring at 200 rpm. A PBS–ethanol solution (60:40, *v*/*v*) [36] was used as the acceptor medium to ensure sink conditions. At specific time points (15 min, 30 min, 45 min, 1 h, 2 h, 4 h, 6 h, 8 h, 12 h), 0.6 mL of the acceptor fluid was collected and immediately replaced with the same amount of fresh medium. Five replicates were performed for each formulation. The amount of drug in the samples was determined using a validated method of High-Performance Liquid Chromatography HPLC (Shimadzu Nexera-i, Kyoto, Japan). The chromatography conditions were as follows: A reversed-phase C18 column (HyperClone BDS C18, 5 µm, 4.6 × 250 mm (Phenomenex, Torrance, CA, USA) was used as the stationary phase. The column temperature was 30.0 ± 0.2 °C. The mobile phase consisted of acetonitrile and 0.05 M potassium dihydrogen phosphate solution, adjusted to pH = 2.9 with 85% orthophosphoric acid, at the ratio of 50:50, respectively. The flow rate was 1.5 mL/min in isocratic mode. The wavelength for agomelatine detection was 230 nm. The statistical analysis was performed using Statistica software, including a U Mann–Whitney test, *p* = 0.05.

### 3.10. Ex Vivo Permeation Studies on Human Cadaver Skin

The study of permeation through human cadaver skin was conducted using Franz diffusion cells (Logan Instruments, Somerset, NJ, USA). The dermatomed human cadaver skin was from the posterior torso part of the body and was obtained from the New York Firefighter Skin Bank (New York, NY, USA). Each Franz diffusion cell was filled with phosphate-buffered saline (PBS) as the acceptor medium, heated to 37 °C, and stirred at 600 rpm. The skin was thawed in PBS at room temperature, mounted on the diffusion cells, and conditioned for 30 min. After this time, about 500 mg of gel was weighed and applied onto the skin using a 1 mL syringe. The donor chambers were covered with parafilm to prevent the sample from drying out during the study. The study lasted for 102 h, and sodium azide 0.02% w/v was added to the acceptor fluid as an antimicrobial agent. At specific time points (4 h, 8 h, 12 h, 24 h, 36 h, 48 h, 60 h, 72 h, 84 h, 96 h, 102 h), 0.3 mL samples were taken and analyzed using High-Performance Liquid Chromatography (HPLC) (Agilent 1100, Agilent Technologies, Santa Clara, CA, USA) and Agilent Chemstation software (OpenLab CDS, ChemStation Edition, Rev. C.01.10, Agilent Technologies. The chromatography conditions were as follows: A reversed-phase C18 column (HyperClone BDS C18, 5 µm, 4.6 × 250 mm (Phenomenex, Torrance, CA, USA) was used. The column was maintained at 30.0 ± 0.2 °C during the analysis. The mobile phase consisted of acetonitrile and 0.05 M potassium dihydrogen phosphate solution, adjusted to pH = 2.9 with orthophosphoric acid 85%, at the ratio of 35:65, respectively. The analysis was run at a 1.0 mL/min flow rate in isocratic mode. The wavelength for AGM detection was 230 nm. The amount of the acceptor medium taken for the analysis was immediately replaced with the fresh portion of the fluid. Six replications were performed for each formulation. After the study, the epidermis and dermis from each skin sample were separated manually using scissors and forceps and inserted in tubes with 3 mm zirconium beads (Benchmark Scientific Inc., Sayreville, NJ, USA). Next, 1 mL of water and ethanol solution (50:50 *v*/*v*) was added to each tube, and the samples were homogenized for 9 min using a BeadBug Microtube Homogenizer (Benchmark Scientific Inc., USA). Then, the samples were centrifuged for 5 min at 10,000 rpm, and the supernatant was analyzed using HPLC to examine the amount of the drug in the tissue. The statistical analysis was performed using Statistica software, including a *t*-test, *p* = 0.05.

## 4. Conclusions

The transdermal administration of agomelatine may provide benefits in increasing bioavailability in the treatment of depression, and the use of microneedles in transdermal delivery could help overcome the *stratum corneum* barrier and potentially result in more effective drug delivery. Both agomelatine hydrogel formulations obtained in this study have the desired rheological and mechanical parameters as a material for coating microneedles in the dip-coating process. Moreover, the formulations were tested for independent application to the skin as transdermal formulations. Both have properties suitable for application to the skin, with the ethanol gel showing better spreadability and skin permeation properties, which may indicate its potentially better effectiveness than the suspension gel.

Further research will include optimizing the method of coating microneedles with both gels in order to compare their behavior during the process and parameters such as film uniformity, dose, and drug release.

## Figures and Tables

**Table 1:** The minimum, maximum, median, and quartile values for length and width measurements of the particles in the Sus AGM gel.

Values [µm]	Length	Width
Minimum	2.5	1.6
Quartile 1 (Q1)	6.3	4.1
Median	8.3	5.4
Quartile 3 (Q3)	10.6	6.9
Maximum	24.2	17.9

**Table 2:** The assignment of selected Raman bands from DFT calculations (B3LYP/6-31G(d,p)) and from the experimental spectrum for AGM.

Theory with Calculation Factor/cm^−1^ [28]	Experimental Data/cm^−1^	Assignment
315		deformation of all molecules
380 383	395	NH b, CH_3_ ab
420	435	CH b oop in benzene ring
430	451	deformation of all molecules
454	472	deformation of methoxy and naphthalene group
498	509	deformation of methoxy and naphthalene group
510	522	deformation of methoxy and naphthalene group
540	555	deformation of all molecules
658	674	b oop of naphthalene group
685	698	deformation of methoxy and naphthalene group oop, CH_2_-CH_2_ t
714		deformation of methoxy and naphthalene group ip
733	735	CH b oop in benzene ring, t CH_2_ r
743	737	CH ab oop in naphthalene group, CH_2_ r
768		CH ab oop in naphthalene group, CH_2_ r
819		CH syb oop in naphthalene group
845	868	b of naphthalene group, b of acetamide group
886	898	CH b oop in benzene ring
959	1003	CH_3_ w in acetamide group, NH bending
983	1018	CC s, CH_2_ t
993	1028	CCs in ethylene group
1032 1047	1060	CH_2_-CH_2_ t CO s in methoxy group, benzene ring r
1062 1074	1080	CN s, CH_3_ sb, CH b in benzene ring CH b ip in benzene ring, CH_2_-CH_2_ t
1120	1130	CH b ip in benzene ring
1145	1182	CH b ip in benzene ring
1164	1187	CH_3_ sb, CH_2_ t, CH b ip in benzene ring
1174	1193	CH_2_ t, CH_3_ ab
1196 1202	1213	CC s, CH_3_ sb, CH b ip in benzene ring, CH_3_-O-C b, CH b ip in benzene ring, CH_2_ t, NH b ip
1226	1250	NH b ip, CH b in CH_2_
1235	1260	CH_2_ w, CH b ip, CH_2_ w in CH_3_, CH b in benzene ring
1247	1268	CO s in methoxy group, CH b ip
1266	1283 1288	CH_2_ w
1289	1304 1311	CH_2_ t
1349 1353	1374	CH_2_ w, CH_3_ symb (umbrella) C=C s between benzene rings, CH_2_ twisting, CH b in benzene ring
1356		C=C as in benzene ring, CH_2_ twisting
1374	1384	C=C as in benzene ring, CH_2_ t
1420		CH_3_ syb (umbrella), CH_2_ sc, CH r in benzene ring
1427 1428 1433	1434	CH_2_ sc, CH_3_ ab, CH_2_ sc, CH_3_ ab, CH_2_ sc, CH_3_ ab, CH b in benzene ring
1442 1444	1446	CH_3_ ab, NH b ip CH_3_ ab, HCCH r in benzene ring
1455	1462	CH_3_ ab, CH r in benzene ring
1460 1462	1472	CH_2_ sc, CH_3_ ab
1486		NH b ip, CH_2_ t, CH_3_ ab
1571	1584	C=C sys in benzene ring
1592		C=C s in benzene ring, CH r in benzene ring
1615	1625	C=C s in benzene ring, CH b ip in benzene ring
1711		C=O s, CH_2_ sc

Description of the type of vibration: s—stretching, b—bending, sc—scissoring, w—waging, t—twisting, r—rocking, ip—in plane, oop—out of plane, a—asymmetrics, sy—symmetrics.

**Table 3:** Comparison of spectral parameters of four selected bands of agomelatine for a temperature of 30 °C and 110 °C.

Raman Shift /cm^−1^	Integral Intensity	Intensity at Point	FWHM /cm^−1^	Raman Shift /cm^−1^	Integral Intensity	Intensity at Point	FWHM /cm^−1^
30 °C				110 °C			
737	39,700	6996	5.33	733	99,254	8226	11.33
1213	20,615	2028	9.55	1210	35,081	2845	11.58
1371	255,992	33,876	7.11	1371	779,001	50,547	14.48
1383	105,843	5682	17.49	1384	80,204	5914	12.74
1584	56,494	6106	8.69	1583	80,519	6959	10.86

**Table 4:** Coefficients calculated from the measurement of the shear stress at a shear rate of 0–300 [1/s].

	Et Placebo 25	Et AGM 25	Sus Placebo 25	Sus AGM 25	Et Placebo 32	Et AGM 32	Sus Placebo 32	Sus AGM 32
K	48.56 ± 1.76	49.23 ± 1.68	98.40 ± 6.26	84.11 ± 1.80	49.90 ± 1.99	50.78 ± 4.11	127.67 ± 9.82	121.30 ± 2.54
n	0.4270 ± 0.0035	0.4242 ± 0.0040	0.3453 ± 0.0085	0.3701 ± 0.0049	0.4132 ± 0.0079	0.4105 ± 0.0095	0.3062 ± 0.0128	0.3166 ± 0.0039

**Table 5:** Yield points calculated from the measurement of deformation depending on the shear stress.

	Et Placebo 25	Et AGM 25	Sus Placebo 25	Sus AGM 25	Et Placebo 32	Et AGM 32	Sus Placebo 32	Sus AGM 32
τ_0_ [Pa]	105.2 ± 2.7	106.2 ± 2.0	279.0 ± 5.9	238.6 ± 22.4	98.0 ± 6.8	100.6 ± 6.4	199.2 ± 10.6	175.0 ± 25.4

**Table 6:** Shear stress values for module intersections.

	Et Placebo 25	Et AGM 25	Sus Placebo 25	Sus AGM 25	Et Placebo 32	Et AGM 32	Sus Placebo 32	Sus AGM 32
τ [Pa] for G′ = G″	189.5 ± 9.3	189.9 ± 4.8	334.4 ± 1.8	372.1 ± 16.0	190.8 ± 0.4	188.6 ± 0.9	369.6 ± 6.3	412.9 ± 6.2

**Table 7:** Values of the coefficients and cumulative amount of drug per cm^2^ permeated in 360 min of study.

	Slope [µg/cm^2^/h]	Linear Part Correlation Efficient R^2^	Cumulative Amount of Drug Released in 720 min [µg/cm^2^]
Et AGM	5.8693	0.9974	1661.74 ± 927.32
Sus AGM	6.1468	0.9989	4413.87 ± 527.52

**Table 8:** Values of the coefficients and the amount of drug permeated and retained in the skin after 102 h of study.

	Jss [µg/cm^2^/h]	Linear Correlation Efficient R^2^	Lag Time [h]	Cumulative Amount of Drug Permeated in 102 h [µg/cm^2^]	Amount of Drug in Epidermis [µg/mg of Tissue]	Amount of Drug in Dermis [µg/mg of Tissue]
Et AGM	30.386	0.9939	37.56	1955.23 ± 247.72	1.457 ± 0.768	0.845 ± 0.241
Sus AGM	2.7491	0.9997	2.48	271.90 ± 43.98	0.205 ± 0.041	0.123 ± 0.032

**Table 9:** Composition of the gels used in the tests [g].

	Et AGM	Et Placebo	Sus AGM	Sus Placebo
Agomelatine	0.5	-	0.5	-
Ethanol 96% *v*/*v*	25.18	25.18	-	-
Glycerol	5.0	5.0	5.0	5.0
Carbopol^®^ EZ-3	0.5	0.5	0.5	0.5
Triisopropanolamine	0.75	0.75	0.75	0.75
Water	18.07	18.57	43.25	43.75

## Data Availability

The original contributions presented in this study are included in this article. Further inquiries can be directed to the corresponding author.
